# The spring stiffness profile within a passive, full-leg exoskeleton affects lower-limb joint mechanics while hopping

**DOI:** 10.1098/rsos.231449

**Published:** 2024-03-20

**Authors:** Stephen P. Allen, Alena M. Grabowski

**Affiliations:** ^1^ Department of Integrative Physiology, University of Colorado Boulder, Boulder, CO, USA; ^2^ Department of Veterans Affairs, Eastern Colorado Healthcare System, Denver, CO, USA

**Keywords:** joint kinetics, power, biomechanics, assistive devices, bouncing gaits

## Abstract

Passive, full-leg exoskeletons that act in parallel with the legs can reduce the metabolic power of bouncing gaits like hopping. However, the magnitude of metabolic power reduction depends on the spring stiffness profile of the exoskeleton and is presumably affected by how users adapt their lower-limb joint mechanics. We determined the effects of using a passive, full-leg exoskeleton with degressive (DG), linear (LN) and progressive (PG) stiffness springs on lower-limb joint kinematics and kinetics during stationary, bilateral hopping at 2.4 Hz. We found that the use of a passive, full-leg exoskeleton primarily reduced the muscle-tendon units (MTUs) contribution to overall joint moment and power at the ankle, followed by the knee, due to the average exoskeleton moment arm around each joint. The greatest reductions occurred with DG springs, followed by LN and PG stiffness springs, probably due to differences in elastic energy return. Moreover, the relative distribution of positive joint power remained unchanged when using a passive, full-leg exoskeleton compared with unassisted hopping. Passive, full-leg exoskeletons simultaneously assist multiple lower-limb joints and future assistive devices should consider the effects of spring stiffness profile in their design.

## 1. Introduction

The spring-like function of the legs has inspired the development of lower-limb passive-elastic exoskeletons to assist human hopping and running, often with the goal of reducing metabolic power [1]. Similar to series-elastic elements like tendons, passive-elastic exoskeletons use springs or other elastic materials to store and return mechanical energy to the user [2,3]. These wearable devices cannot produce net positive work but are able to provide a portion of the overall joint moment and power during hopping and running so that the contribution of the muscles and tendons, or muscle-tendon units (MTUs), surrounding each joint is reduced [3,4]. In turn, the use of a passive-elastic exoskeleton can reduce whole-body metabolic power by decreasing the required muscle force [4] and active muscle volume [1,5] compared with hopping or running without an exoskeleton.

Previous research on exoskeleton has often assisted the lower limb joint that generates the greatest positive mechanical power during either the ground contact [3,6] or swing phases [7,8] of level-ground hopping and running. Positive rather than negative mechanical power is targeted because producing force from concentric muscle contractions is less efficient than from eccentric muscle contractions. While experimental measurements cannot separate muscle and tendon contributions *in vivo*, the combined MTUs surrounding the ankle and knee joints provide 53–80% and 18–46% of the total positive mechanical power generated by the leg while hopping without an exoskeleton from 2.2 to 3.2 Hz, respectively [3]. Similarly, when running without exoskeletal assistance at 2.0–3.25 m s^−1^, the MTUs surrounding the ankle, knee and hip joint account for 42–47%, 19–21% and 32–39% of the total positive power over a stride, respectively [9]. Thus, several passive-elastic exoskeletons have been designed to assist at the ankle joint during the ground contact phase of hopping and running [3,6,10] or at the hip joint during the swing phase of running [7,8]. For example, Farris and colleagues showed that hopping in place at 2.5 Hz with a passive, ankle-only exoskeleton with linear (LN) springs reduced metabolic power by 13% [3,4]. The exoskeleton also reduced the MTU average plantarflexor moment by 30–50%, so that the positive ankle power supplied by biological tissues was decreased by approximately 50% while users were able to maintain similar overall leg stiffness compared with hopping without an exoskeleton [3,4]. However, despite adapting to exoskeletal assistance and reducing musculoskeletal demands [3,6,10], reductions in metabolic power are not guaranteed for hopping and running [6,11,12].

Passive-elastic exoskeleton designs that act during the ground contact phase of hopping and running may need to simultaneously assist multiple joints to achieve the greatest reductions in muscle force, active muscle volume and metabolic power [2,3,13,14]. While the MTUs surrounding the ankle contribute the most to total positive mechanical power during hopping and running, the MTUs that surround the knee and hip joints also significantly contribute to total positive mechanical power during hopping and running. Previously, Farris and colleagues [3,4,15] found that hopping with a passive-elastic ankle-only exoskeleton resulted in small mechanical changes to the knee, where 6% of the total positive power was redistributed from the knee to the ankle and almost half of the reduction in metabolic power could be attributed to this change. Proximal leg muscles that surround the knee and hip joints are thought to be less efficient than distal leg muscles such as the plantarflexor muscles [16,17]. Thus, an exoskeleton that spans the ankle, knee and hip joints may further reduce the metabolic power of hopping and running by providing greater assistance to the knee or hip compared with an exoskeleton that only spans one joint.

Previously, Grabowski and Herr [2] found that a passive-elastic full-leg exoskeleton (which crossed the ankle, knee and hip joints) reduced the metabolic power of stationary hopping by 18–28% across frequencies of 2.2–2.8 Hz and users reduced their biological leg stiffness to maintain an overall leg stiffness similar to hopping without an exoskeleton [2]. However, it is unknown how users adjusted their lower-limb joint mechanics while hopping with the assistance of the full-leg exoskeleton and if there is a redistribution of total positive power among the lower-limb joints. This information may provide valuable insight for the future design of passive-elastic and powered exoskeletons to augment hopping and running performance.

Hopping performance while using a passive-elastic exoskeleton also depends on the elastic properties of the springs within the device. For example, springs that are too stiff in stationary hopping could decrease the metabolic benefits provided by a passive ankle-only or full-leg exoskeleton by changing the hopping frequency or compromising balance [2,18]. In addition, changes in metabolic power when using a passive-elastic exoskeleton have been shown to depend on a spring’s stiffness profile, which refers to the continuous slope of the force–displacement curve [12]. Previous research on passive-elastic ankle-only exoskeletons used a linear-tension spring during hopping and running, where stiffness is constant throughout the spring’s displacement [3,4,6,15,18]. However, experiments using a passive-elastic, full-leg exoskeleton during stationary hopping have demonstrated that changes in metabolic power depend on the spring’s stiffness profile and the amount of elastic energy stored and returned, despite the springs exhibiting the same average stiffness for a given displacement [12]. Compared with hopping without an exoskeleton at 2.4 Hz, the use of a passive-elastic full-leg exoskeleton with degressive (DG) stiffness springs (initially stiff but becomes less stiff with compression) and linear (LN) stiffness springs decreased metabolic power by 24% and 16%, respectively, while the use of progressive (PG) stiffness springs (initially compliant but stiffness increases nonlinearly with compression) did not significantly affect metabolic power [12].

Users were able to maintain consistent spring-mass mechanics with each spring stiffness profile, where there were no changes in ground contact time, hop height, or overall leg stiffness compared with hopping without an exoskeleton (NH). However, other measures, such as the centre of mass displacement during the ground contact phase and peak vertical ground reaction force, were reduced while hopping using a passive-elastic full-leg exoskeleton with DG or LN stiffness springs, which indicates that the spring stiffness profile may elicit different effects on lower-limb joint angles, moments and powers compared with hopping without an exoskeleton.

The design of the passive-elastic full-leg exoskeleton includes springs that span the entire leg, attaching to a waist harness with mounts near the hip joint centre of rotation and shoe mounts near the metatarsal joint centre of rotation in the sagittal plane. Presumably, this design primarily affects the MTUs surrounding the ankle and knee, given that the exoskeleton’s sagittal plane moment arm around the hip joint is designed to be smaller than that at the ankle or knee joint and those surrounding the hip joint provide a negligible contribution to total leg power during hopping without an exoskeleton [3]. The influence of using a full-leg exoskeleton with different spring stiffness profiles on joint mechanics may provide further insight into the changes in metabolic power during bouncing gaits and inform future designs of passive-elastic exoskeletons.

We determined the effects of hopping with and without using a passive-elastic full-leg exoskeleton with three different spring stiffness profiles on lower-limb joint mechanics. We hypothesized that peak flexion angles and the range of motion at the ankle, knee and hip joints would decrease while hopping using the passive-elastic full-leg exoskeleton compared with hopping without an exoskeleton. We also hypothesized that hopping using a passive-elastic full-leg exoskeleton would reduce the MTU joint moment and positive power compared with hopping without the exoskeleton, with the greatest reductions occurring with the DG stiffness springs, followed by LN and PG stiffness springs, respectively. Finally, we hypothesized that hopping using a passive-elastic full-leg exoskeleton would redistribute overall positive joint power away from the knee to the ankle, compared with hopping without the exoskeleton.

## 2. Materials and methods

### 2.1. Participants

Fourteen individuals (table 1) with no reported cardiovascular, neurological, or musculoskeletal impairments participated in the study. All participants self-reported exercising for at least 30 min per day at or above a moderate intensity, three times per week, for the last six months and provided written informed consent according to the University of Colorado Boulder Institutional Review Board.

**Table 1.** Participant characteristics and exoskeleton stiffness organized by body mass.

sex	age (yr)	height (m)	leg length (m)	mass (kg)	k_calc_ (kN m^−1^)	k_DG_ (kN m^−1^)	k_LN_ (kN m^−1^)	k_PG_ (kN m^−1^)
F	37	1.70	0.92	58.9	7.84	7.8	7.00	8.00
F	22	1.53	0.86	60.0	7.98	8.4	8.44	8.00
F	23	1.70	0.87	60.6	8.08	7.8	8.44	8.00
F	25	1.65	0.87	62.0	8.24	7.8	8.44	8.00
F	26	1.73	0.93	62.8	8.36	8.2	8.44	8.00
F	26	1.62	0.84	62.9	8.38	7.8	8.44	8.00
F	23	1.59	0.85	63.6	8.46	7.8	8.44	8.00
M	43	1.63	0.94	65.4	8.70	8.2	8.44	8.00
M	20	1.67	0.90	65.9	8.80	9.0	8.44	9.00
M	24	1.73	0.94	67.5	8.98	8.2	8.44	9.00
M	30	1.79	0.94	69.2	9.22	8.2	9.46	9.00
M	25	1.80	0.98	71.6	9.50	9.4	9.46	10.00
M	20	1.64	0.86	75.3	9.96	10.6	9.46	10.00
M	30	1.73	0.88	77.7	10.34	10.6	10.6	10.00
Avg.	26.7	1.68	0.90	66.0	8.77	8.56	8.71	8.64
s.d.	6.5	0.08	0.04	5.7	0.75	0.99	0.82	0.84

*Notes.* The leg length was measured from the greater trochanter to the floor while standing; k_calc_ is the calculated exoskeleton stiffness for one leg; k_DG_ is the degressive spring stiffness; k_LN_ is the linear spring stiffness; k_PG_ is the progressive spring stiffness. All stiffness values represent the sum of both springs and were calculated for 10 cm of displacement. Differences in k_DG_ relative to mass are due to variations in the leg length.

### 2.2. Exoskeleton design

**Figure 1.**
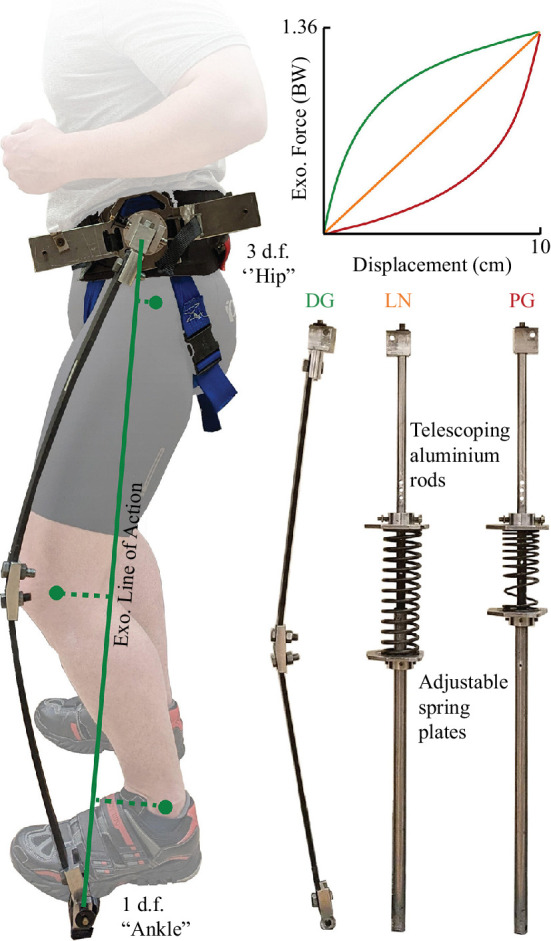
Illustration of the exoskeleton (Exo.) line of action (solid green line) relative to the lower-limb joints. The exoskeleton moment arms relative to each joint (green filled circle) for a discrete time point are indicated by dashed lines and represent the perpendicular distance from the Exo. line of action to the centre of each joint. The line of action was determined from reflective markers placed on the hip and foot attachment points of the exoskeleton and are nt pictured. The Exo included a 3 degree-of-freedom (d.f.) hip attachment point and a 1 d.f. ankle attachment point. We used three spring stiffness profiles within the exoskeleton that had the same discrete stiffness for 10 cm of displacement, where the force of the exoskeleton is indicated in units of body weight (BW) and the stiffness profiles were DG (green), LN (orange) and PG (red).

A detailed description of the full-leg exoskeleton construction can be found in previous publications [2,12]. In brief, the full-leg exoskeleton design consists of a waist mount, foot mounts on each shoe and two exoskeleton ‘legs’. Each leg incorporates a spring and compresses in parallel with the legs during the stance phase of stationary, bilateral hopping (figure 1). The waist mount comprised an adjustable aluminium frame that is secured tightly around the user’s waist with a padded hip belt and nylon webbing harness that wraps around the thighs. This frame includes a 3 degree-of-freedom (d.f.) joint (flexion/extension, abduction/adduction and internal/external rotation) positioned near the hip joint’s centre of rotation in the sagittal plane. The foot mounts comprised aluminium pin joints secured to a steel plate that is fastened to the bottom of clipless mountain biking shoes via the cleat mounting holes (Gavin MTB Cycling Shoe, Elkton, FL or Trace MTB Shoe, Diamondback, Kent, WA). These pin joints allow 1 d.f. (plantar/dorsiflexion) and are positioned near the metatarsal-phalangeal joint’s centre of rotation in the sagittal plane. The exoskeleton ‘legs’ connect the waist harness and foot mounts and act in parallel with the user’s legs.

We used three sets of springs with different stiffness profiles (figure 1). Custom-made fibreglass leaf springs (GC067 UB; Gordon Composites, Montrose, CO) were used to create the DG stiffness spring, which is characterized by a high initial stiffness that becomes less stiff with further compression [2]. The leaf springs consisted of ‘thigh’ and ‘shank’ segments that varied in length by increments of 2.54 cm and the ‘thigh’ segment was always 2.54 cm longer than the ‘shank’ segment. These segments were joined at a 165° angle using a piece of aluminium to ensure that the leaf springs would always bend anteriorly towards the user. We used metal compression springs with even pitch coils for the LN stiffness springs, and variable pitch coils were used for the PG stiffness springs, which are characterized by a low initial stiffness that increases exponentially with compression [12]. These compression springs were placed between two aluminium plates that could be adjusted along the length of two telescoping rods to accommodate the coiled springs of different lengths and diameters (figure 1). When the telescoping rods were attached to the waist harness and foot mounts, the springs could be compressed by the aluminium plates during the stance phase.

We measured the stiffness of each fully assembled exoskeleton ‘leg’ prior to the experimental tests using a materials testing machine to measure instantaneous force and displacement (Series 5859; Instron, Norwood, MA) [12]. Because of the nonlinear stiffness profiles of the DG and PG springs, we characterized the stiffness for all of the springs at 10 cm of compression, which approximates the average centre of mass displacement while hopping without an exoskeleton at 2.4 Hz [2,12,19]. We provided participants with springs of the same body mass-normalized stiffness (approx. 0.132 kN m^−1^ kg^−1^ for both springs) at 10 cm of compression (table 1), which was previously determined to provide the greatest reduction in metabolic power compared with hopping without an exoskeleton at 2.4 Hz [12]. A detailed explanation of how this mass-normalized stiffness was calculated can be found in previous papers [2,12].

We sized each exoskeleton to fit the participant while their ankles were plantarflexed, with knees and hips extended, so that they were standing on their toes. We measured the distance from the greater trochanters of each participant to the floor under the metatarsal heads to determine the exoskeleton ‘leg’ length. This allowed the compression of the springs to begin at the instant of ground contact. For the DG stiffness springs, we selected fibreglass segments to achieve the same approximate exoskeleton ‘leg’ length when the spring was fully assembled. The stiffness of the DG springs is affected by their length, with longer leaf springs being more compliant and shorter ones are stiffer for the same thickness. We controlled this effect by increasing or decreasing the thickness of the fibreglass by increments of approximately 0.05 cm to achieve a spring with stiffness as close as possible to the body mass-normalized stiffness at 10 cm of compression. When affixed to the waist harness and foot mounts, the telescoping rods for the LN and PG stiffness springs were able to match the measured exoskeleton ‘leg’ length by sliding past each other. Pre-drilled holes on the ‘thigh’ segment spaced approximately 1.27 cm apart allowed us to set the height of the aluminium plates to accommodate different lengths of coil springs and ensure that compression began at the instant of ground contact.

### 2.3. Experimental protocol

We simultaneously measured ground reaction forces (GRFs) and lower body kinematics while participants hopped in place at 2.4 Hz under four conditions: normal bipedal hopping without an exoskeleton (NH) and using passive-elastic full-leg exoskeletons with DG, LN and PG stiffness springs. Each trial was 2 min long and we analysed 20 hops from the final 30 s of each trial.

Participants hopped in place on both legs on a stationary, split-belt treadmill (Bertec, Columbus, OH; 1000 Hz) equipped with a force plate under each foot so that separate GRF vectors could be attributed to each leg. Lower body kinematics were recorded using a 10-camera motion capture system (Vicon, Centennial, CO; 200 Hz) with a total of 38 reflective markers placed on the pelvis and both legs. We placed markers on the skin or form-fitted clothing at the anterior and posterior iliac spines, the superior aspect of the iliac crests, greater trochanters, bilaterally on the femoral condyles and malleoli, and on the shoes at the first and fifth metatarsal heads and the posterior aspect of the calcaneus. Rigid clusters of four markers were placed on the thigh and shank segments and secured with soft Velcro wraps. When participants hopped using the exoskeleton, the padded waistbelt covered the pelvis, so we placed markers on the outside of the belt at our best estimate of the pelvis anatomical landmarks. Additionally, we placed markers on the exoskeleton hip and shoe attachments to measure spring displacement and orientation.

Participants were given a short acclimatization period (1–3 min) to hop at 2.4 Hz prior to each condition until they reported feeling comfortable using each exoskeleton and the research team visually confirmed the participants’ ability to match the target hopping frequencies consistently while maintaining an aerial phase between hops. We randomized the order of conditions and used an audible metronome to ensure the hopping frequency. Participants were given at least 2 min of rest between trials due to donning, doffing and assembly of the exoskeleton ‘legs’.

### 2.4. Data analysis

We labelled reflective marker trajectories (Vicon Nexus, Oxford, UK) and exported them to Visual 3D (C-motion, Germantown, MD) with GRFs for inverse dynamics analysis. We used a fourth-order, low-pass Butterworth filter with a 20 Hz cut-off [20] for both marker and GRF data and used a 20 N vertical GRF threshold to determine ground contact events. We calculated sagittal plane joint angles, moments and powers for the ankle, knee and hip. We measured joint angles as the inter-segment angle between proximal and distal segments, where an increase in joint angle represents extension or plantarflexion (90° ankle angle is equivalent to the anatomical reference position) and range of motion was determined as difference between the maximum and the minimum joint angles throughout the hop cycle. We calculated the overall joint moment using joint angles and GRFs (including the weight of the exoskeleton) and resolved joint moments in the proximal segment’s coordinate system. We then calculated overall joint power as the dot product of the overall joint moment and angular velocity.

Under the exoskeleton conditions, a portion of the overall joint moment and power was provided by the exoskeleton, while the rest was supplied by the MTUs. To estimate the exoskeleton contributions to overall joint moment and power in the sagittal plane, we followed the process and methods outlined in two previous studies [13,14]. We first assumed that there was no soft-tissue artefact and estimated the instantaneous exoskeleton force for each leg. We calculated the instantaneous linear displacement of the springs from reflective markers placed on the hip and shoe attachments of the exoskeleton and then used the *a priori* measured force–displacement data from the materials testing machine to estimate the instantaneous exoskeleton spring force, assuming that the exoskeleton was rigidly attached and accounting for energy loss due to hysteresis [12]. Because the exoskeleton attachments are low friction pin joints that allow rotation and approximately 99% of the resultant GRF is directed vertically in hopping [21], we also used the reflective markers on the exoskeleton attachments to define the line of action for the exoskeleton force (figure 1). We then determined the exoskeleton moment arms about each joint as the perpendicular distance from each joint centre to the exoskeleton line of action (figure 1). The exoskeleton contribution to the overall joint moment for each joint was estimated as the cross-product of the exoskeleton moment arm relative to each joint and the estimated exoskeleton force, resolved in the proximal segment coordinate system [22]. Then, we estimated the exoskeleton contribution to overall joint power by multiplying the estimated exoskeleton moment by the joint’s angular velocity. We derived the MTU contribution to overall joint moment and power as


(2.1a) $$M_{j,mtu}=M_{j,overall}-M_{j,exo},$$

(2.1b) $$P_{j,mtu}=P_{j,overall}-P_{j,exo},$$

where *j* represents a lower-limb joint, and *M* and *P* represent moment and power, respectively.

We considered moments that acted to extend the joints or plantarflex the ankle to be positive.

At each joint, we separately calculated the MTUs’ and exoskeleton’s contributions to total average positive mechanical power according to Farris and Sawicki [3]. First, we used the trapezium method for integrating positive power over the hop cycle and then summed all periods of positive work to determine total positive work. Next, total positive work was divided by the duration of the hop cycle to determine the average positive power over the hop cycle. The average positive power could then be summed across each joint to estimate the total average positive power ( ${\bar{P}}_{tot}$ ),


(2.2) $${\bar{P}}_{tot}={\bar{P}}_{ankle,overall}+{\bar{P}}_{knee,overall}+{\bar{P}}_{hip,overall},$$

where ${\bar{P}}_{ankle}$ , ${\bar{P}}_{knee}$ and ${\bar{P}}_{hip}$ are the average positive powers from the ankle, knee and hip joints, respectively.

The distribution of total average positive power could then be expressed as a percentage (*P*%) of total average positive power,


(2.3) $$P\%=\frac{{\bar{P}}_{j,c}}{{\bar{P}}_{tot}}\cdot 100\%,$$

where ${\bar{P}}_{j,c}$ is the average positive power from any joint and contribution (MTU or exoskeleton). We performed all kinetic calculations separately for each leg before summing the two legs together.

### 2.5. Statistical analysis

We performed a repeated-measures analysis of variance (ANOVA) in R (v. 4.2.2, [23]) using the nlme [24] and ANOVA [23] packages to determine the effect of the hopping condition on peak joint flexion and range of motion, the MTU average moment and power at each joint and the distribution of overall average positive power between the ankle, knee and hip joints. We considered the hopping conditions as a fixed categorical effect and participants as a random effect. If a significant main effect of the hopping condition was found, we performed *post hoc* paired *t*-tests with a Sidak correction for multiple comparisons (α = 0.05) using the emmeans package [25]. Although not a part of our hypotheses, we also performed the same analysis on hopping frequency, ground contact time, peak vertical ground reaction force (vGRF; summed from the two plates) and hop height (calculated according to Cavagna [26]), to determine if participants performed the same overall hopping task with the full-leg exoskeleton compared with NH.

## 3. Results

We found that participants maintained a consistent hopping frequency (F_3,39_ = 1.0 and *p* = 0.30) and ground contact time (F_3,39_ = 1.38 and *p* = 0.26) across all conditions (table 2). However, there was an effect of hopping condition on peak vGRF (F_3,39_ = 7.96 and *p* < 0.001), where hopping using a full-leg exoskeleton with PG stiffness springs increased peak vGRF by 12.2% (271 ± 131 N [avg. ± s.d.] and *p* < 0.01) compared with NH and 14.3% (276 ± 333 N and *p* < 0.001) compared with using an exoskeleton with DG stiffness springs. We also found an effect of condition on hop height (F_3,39_ = 3.024 and *p* = 0.04), with no significant differences between NH and any of the exoskeleton conditions (*p* ≥ 0.42) but hopping using a full-leg exoskeleton with DG stiffness springs increased hop height by 31.6% (0.7 ± 1.1 cm and *p* = 0.037) compared with with PG stiffness springs.

**Table 2.** Hopping kinematics and kinetics.

condition	frequency (Hz)	contact time (s)	peak vGRF (N)	hop height (cm)
NH	2.40 ± 0.01	0.26 ± 0.03	2253 ± 289	3.1 ± 1.0
DG	2.40 ± 0.01	0.25 ± 0.03	2248 ± 380^a^	3.4 ± 1.2
LN	2.41 ± 0.01	0.26 ± 0.03	2387 ± 287	2.9 ± 1.1
PG	2.41 ± 0.01	0.26 ± 0.03	2524 ± 310^b^	2.8 ± 1.0^a^

*Notes.* Group means ± s.d. vGRF: vertical ground reaction force. Conditions are NH and hopping using an exoskeleton with DG, LN and PG stiffness springs.

^a^ Significant difference (*p* < 0.05) from DG.

^b^ Significant difference (*p* < 0.05) from NH.

### 3.1. Joint angles

**Figure 2.**
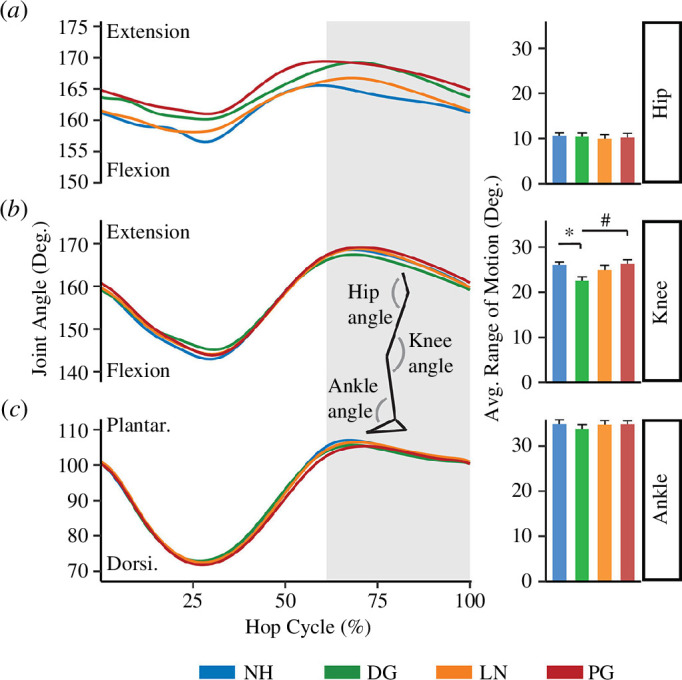
Average joint angle and range of motion (±s.e.) for the hip (*a*), knee (*b*) and ankle (*c*) during NH (blue) and hopping with an exoskeleton using DG (green), LN (orange) and PG (red) stiffness springs. Data are time normalized over the hop cycle and represent the average of both legs. The shaded area represents the average aerial phase across all conditions. * indicates that range of motion was significantly different (*p* < 0.05) from NH and # indicates that range of motion was significantly different (*p* < 0.05) from DG.

We found no significant difference in the peak flexion angle between any condition at the ankle (figure 2; F_3,39_ = 0.522 and *p* = 0.70), knee (F_3,39_ = 1.672 and *p* = 0.19) or hip joints (F_3,39_ = 2.683 and *p* = 0.06). Additionally, we found no significant difference in ankle or hip joint range of motion between any condition (F_3,39_ = 1.380 and *p* = 0.26; F_3,39_ = 0.272 and *p* = 0.85, respectively). However, there was a difference in knee joint range of motion (F_3,39_ = 9.644, *p* < 0.001), where hopping using a full-leg exoskeleton with DG stiffness springs decreased knee joint range of motion by 10.5% (3.5 ± 3.0^o^, *p* < 0.01) compared with NH and 11.2% (3.8 ± 2.9^o^, *p* < 0.01) compared with an exoskeleton with PG stiffness springs.

### 3.2. Joint moments

**Figure 3.**
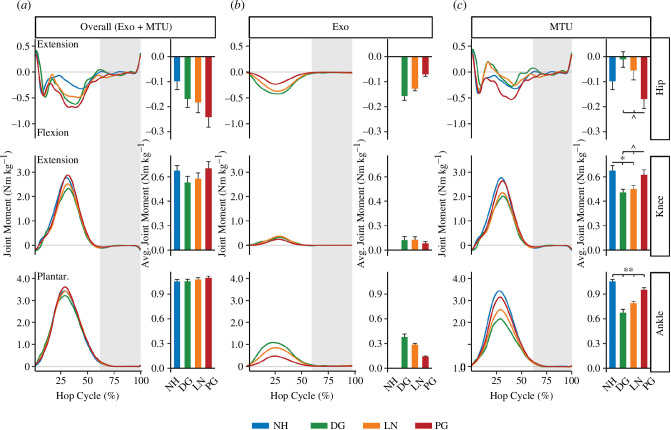
Instantaneous and cycle-average joint moment (±s.e.). Overall (*a*) is the sum of the exoskeleton (Exo; *b*) and MTU (*c*) contributions to joint moment for the hip (top row), knee (middle row) and ankle (bottom row). Colours represent the different conditions of NH (blue) and DG (green), LN (orange) and PG (red) stiffness springs. Joint moment is normalized to biological mass and represents both legs. The shaded grey region represents the average aerial phase across all conditions. ** indicates that joint moment was significantly different (*p* < 0.05) for all conditions, * indicates that joint moment was significantly different (*p* < 0.05) from NH, ^ indicates that joint moment was significantly different (*p* < 0.05) from PG. Note the different y-axis scales for each joint.

While hopping using a full-leg exoskeleton, participants decreased the MTU contribution to the overall average joint moment at the ankle (figure 3; F_3,39_ = 90.927 and and p < 0.001) and 20, knee (F_3,39_ = 21.724 and *p* < 0.001) and hip (F_3,39_ = 8.113 and *p* < 0.001). We found that hopping using a full-leg exoskeleton with DG stiffness springs reduced the MTU average ankle plantarflexion moment by 36% (0.38 ± 0.13 Nm kg^−1^ and *p* < 0.0001) and average knee extension moment by 27.4% (0.18 ± 0.11 Nm kg^−1^ and *p* < 0.0001) compared to NH. Similarly, hopping using a full-leg exoskeleton with LN stiffness springs reduced the MTU average ankle plantarflexion moment and knee extension moment by 24.0% (0.26 ± 0.05 Nm kg^−1^ and *p* < 0.0001) and 22.4% (0.15 ± 0.12 Nm kg^−1^ and *p* < 0.0001) compared with NH. Finally, hopping using a full-leg exoskeleton with PG stiffness springs reduced the MTU average ankle plantarflexion moment by 9.4% (0.10 ± 0.04 Nm kg^−1^ and *p* < 0.01) compared with NH.

### 3.3. Joint powers

**Figure 4.**
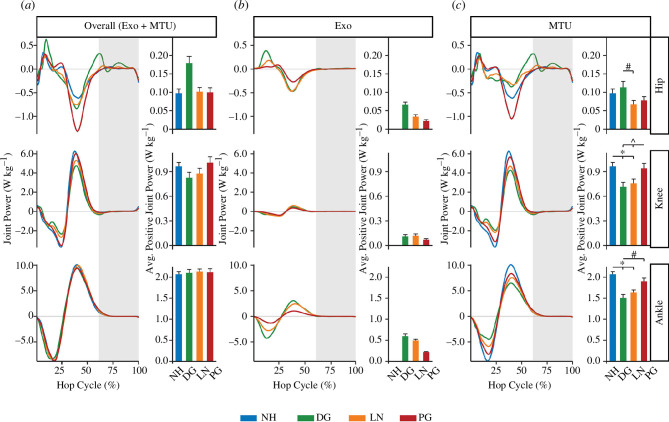
Instantaneous and cycle-average positive joint power (±s.e.). Overall (*a*) is the sum of Exo (*b*) and MTU (*c*) contributions to joint power for the hip (top row), knee (middle row) and ankle (bottom row). Colours represent the different conditions of NH (blue) and DG (green), LN (orange) and PG (red) stiffness springs. Joint power is normalized to biological mass and represents both legs. The shaded grey region represents the average aerial phase across all conditions. * indicates that joint power was significantly different (*p* < 0.05) from NH, # indicates that joint power was significantly different (*p* < 0.05) from DG and ^ indicates that joint power was significantly different (*p* < 0.05) from PG. Note the different y-axis scales for each joint.

We found that there was no significant difference in total average positive power between conditions (F_3,39_ = 1.029 and *p* = 0.39). Hopping using a full-leg exoskeleton resulted in significant reductions in the MTU average positive power at the ankle and knee joints (figure 4; F_3,39_ = 22.05 and *p* < 0.001 and F_3,39_ = 10.46 and *p* < 0.001, respectively). Compared with NH, we found that hopping using a full-leg exoskeleton with DG or LN stiffness springs decreased the MTU average positive ankle power by 26.6% (0.57 ± 0.30 W kg^−1^ and *p* < 0.0001) and 19.5% (0.43 ± 0.13 W kg^−1^ and *p* < 0.0001), respectively. Additionally, the use of the exoskeleton with the DG and LN stiffness springs decreased the MTU average positive knee power by 24.6% (0.25 ± 0.23 W kg^−1^ and *p* < 0.001) and 20.0% (0.21 ± 0.23 W kg^−1^ and *p* < 0.01), respectively, compared with NH. We also found an effect of condition on the MTU average positive hip power (F_3,39_ = 4.22 and *p* = 0.011). However, pairwise comparisons revealed that the MTU average positive hip power was not different for any of the exoskeleton conditions compared with NH (*p* ≥ 0.22). Instead, we found that the MTU average positive hip power increased by 48.7% (0.03 ± 0.03 W kg^−1^ and *p* = 0.004) when using an exoskeleton with DG stiffness springs compared with LN stiffness springs. The use of the PG stiffness springs did not significantly reduce the MTU average positive power compared with NH for any lower-limb joint (*p* ≥ 0.12).

**Figure 5.**
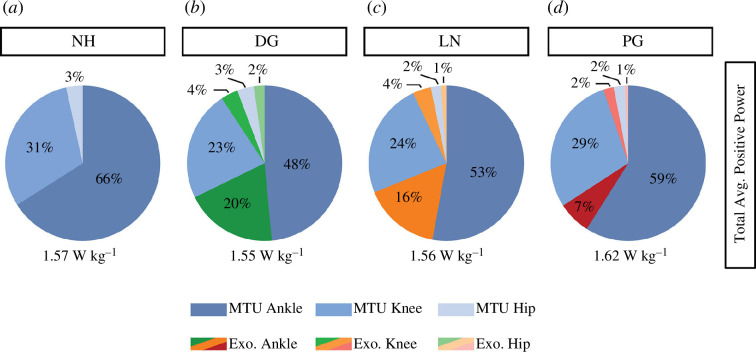
Relative joint contributions to total average (Avg.) positive power during NH (*a*), DG (*b*), LN (*c*) and PG (*d*) stiffness springs. Total average positive power is displayed below each chart and normalized to biological mass.

The distribution of total average positive power was nearly constant across all conditions (figure 5). We found that participants did not change the relative proportion of overall average positive ankle power to total positive leg power (figure 5, F_3,39_ = 0.282 and *p* = 0.84). There was an effect of condition on the relative proportion of overall average positive knee power to total positive leg power (F_3,39_ = 3.56 and *p* = 0.03) but the exoskeleton conditions were not significantly different from NH (*p* ≥ 0.17). There was also an effect of condition on the relative proportion of overall average positive hip power to total positive leg power (F_3,39_ = 12.12 and *p* < 0.001), where hopping using a passive-elastic full-leg exoskeleton with DG stiffness springs increased the relative proportion of overall average positive hip power from 3% to 5% compared with all other conditions (*p* ≤ 0.0001).

## 4. Discussion

In this study, we quantified the joint-level mechanics of bilateral, stationary hopping at 2.4 Hz while using a passive-elastic, full-leg exoskeleton with three different spring stiffness profiles: DG, LN and PG (figure 1), compared with NH. Overall, our findings indicate that the use of a passive-elastic full-leg exoskeleton primarily assists the ankle and knee by reducing the MTU average joint moment and power compared with NH. However, the spring stiffness profile affects the magnitude of assistance, with the lowest MTU average joint moment and power observed using an exoskeleton with DG stiffness springs, followed by LN and PG stiffness springs, respectively.

Our data partially support our first hypothesis, which posited that hopping using a passive-elastic full-leg exoskeleton with any spring stiffness profile would decrease peak flexion angles and range of motion at the ankle, knee and hip compared with NH. We found that peak flexion angles at each joint were not significantly different from NH when hopping using a passive-elastic full-leg exoskeleton with any of the three spring stiffness profiles (figure 2). However, we also found that the use of an exoskeleton with DG stiffness springs reduced the knee range of motion compared with all other conditions (figure 2). The reduction in the knee range of motion with DG stiffness springs was small (approx. 4°) but is probably attributed to reductions in the peak knee extension angle during the aerial phase and may be indicative of the design of the exoskeleton with DG stiffness springs. The DG stiffness springs were singular springs with a fixed resting length and spanned the entire leg (figure 1; table 1). We sized each spring to the participants so that the spring would begin to compress at the instant of ground contact. However, the spring may have applied compression forces to the leg during toe-off or the start of the aerial phase if the resting length of the spring was not long enough. Participants may have reduced their knee joint range of motion to prevent the DG stiffness springs from stretching beyond their resting length and absorbing some of the positive power needed to achieve an aerial phase. Alternatively, if the resting lengths of the DG stiffness springs were set too long, we observed that participants had difficulty maintaining balance or achieving the target hopping frequency during the acclimatization period before the experimental trial. In contrast, the LN and PG stiffness springs were mounted between adjustable aluminium plates on telescoping rods so that the exoskeleton ‘leg’ length always matched the participant’s leg length at any point in the hop cycle. The LN and PG stiffness springs were not fixed to the aluminium plates, which allowed the springs to be compressed during the stance phase but never stretched beyond their resting length in the aerial phase. This design probably allowed participants to have the same range of motion as the NH condition. These results add to the previous literature suggesting that a spring’s resting length should be optimized for the participant on an individual basis [3,10,11] and that studies using an exoskeleton spring that spans the leg should strive to match the resting spring length to a participant’s extended leg length so that elastic energy stored in the spring during compression is not lost to stretching the spring beyond its resting length in the aerial phase.

Our findings support our second hypothesis that hopping using a passive-elastic full-leg exoskeleton would decrease MTU average joint moment and power compared with NH, with the greatest reductions occurring with the DG stiffness springs, followed by the LN and PG stiffness springs, respectively. We found that hopping using a passive-elastic full-leg exoskeleton reduced the MTU average ankle plantarflexor moment by 36%, 24% and 9% using the DG, LN and PG stiffness springs, respectively. Additionally, the use of the exoskeleton with the DG and LN stiffness springs decreased the MTU average knee extensor moment by 24–27%, while the PG stiffness springs did not (figure 3). Ultimately, these changes allowed participants to reduce the MTU average positive joint power, with the largest reductions at the ankle then the knee (figures 4 and 5). We also found that hopping using a full-leg exoskeleton with the DG stiffness springs provided 26% of the total average positive power of the leg (figure 5), while the LN and DG stiffness springs provided 19% and 10%, respectively. The differences in total average positive power between spring stiffness profiles are probably due to the magnitude of elastic energy that can be stored and returned by the spring for a given displacement [12]. For example, for a 10 cm displacement, a DG stiffness spring can store 36% more elastic energy per hop than an LN stiffness spring and 186% more than a PG stiffness spring [12].

**Figure 6.**
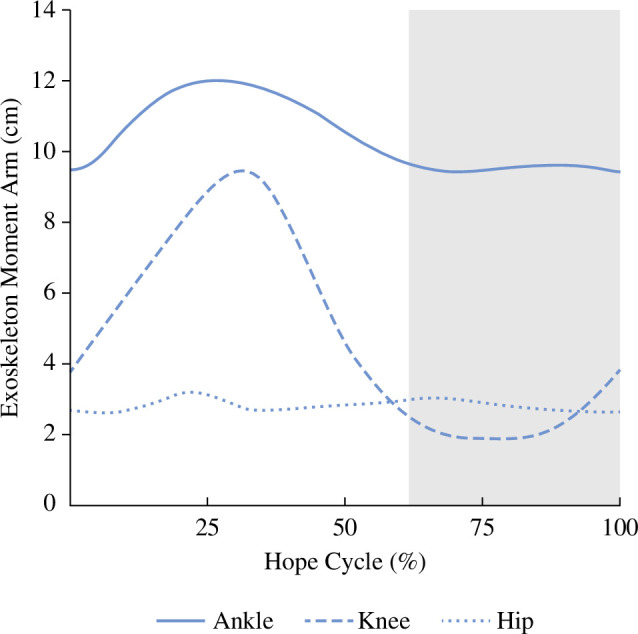
Average sagittal-plane exoskeleton moment arms while hopping using the passive-elastic, full-leg exoskeleton. The instantaneous moment arms are equal to the perpendicular distance from the line of action of the exoskeleton to the ankle, knee and hip joint centres. Data are averaged across DG, LN and PG stiffness springs. The grey area represents the average aerial phase across all exoskeleton conditions.

The full-leg exoskeleton had the greatest influence on the ankle due to the average length of the exoskeleton moment arm throughout the stance phase. The attachment points of the exoskeleton ‘leg’ are placed close to the hip and metatarsal heads, with the pin joints allowing for a direct line of action between them. As the joints of the lower limb flex and extend throughout the stance phase, the ankle and knee joint centres move relative to the exoskeleton’s line of action. We found that the longest average exoskeleton moment arm in the sagittal plane was at the ankle, followed by the knee and hip, respectively, despite the knee having the largest range (figure 6). We also observed that the exoskeleton provided a flexion moment around the hip (figure 3), suggesting that the exoskeleton’s line of action was oriented at a slight angle that acts posterior to the hip joint centre in the sagittal plane. However, despite this observation, we did not translate to any significant changes in positive power at the hip compared with NH.

Finally, our data do not support our hypothesis that hopping using a full-leg exoskeleton would redistribute overall joint power away from the knee to the ankle. Similar to hopping with a passive-elastic, ankle-only exoskeleton with LN stiffness springs [3], humans adopt a strategy to maintain the distribution of joint power between normal hopping and hopping with a passive-elastic, full-leg exoskeleton using the DG, LN or PG stiffness springs. Across all conditions, we found that the ankle and knee joints provided approximately 66–69% and 26–31% of the total average positive power of the leg, respectively, with the remainder coming from the hip (figure 5). Presumably, maintaining the distribution of average positive power output at each joint allowed participants to hop with constant ground contact times, peak vGRFs and hop heights while hopping using a passive full-leg exoskeleton with the DG and LN stiffness springs (table 2). We observed an increase in peak vGRF and a decrease in hop height while using the exoskeleton with the PG stiffness springs, which is consistent with our previous study [12]. This probably occurred because the PG stiffness springs cannot store and return enough elastic energy to offset the total weight of the device and is reflected by the increase in the total average positive power output (figure 5).

Contrary to our expectations, using a passive-elastic full-leg exoskeleton did not result in greater total positive power contributions compared with an ankle-only exoskeleton during hopping. We found that the full-leg exoskeleton with the LN stiffness springs provided 21% of the total positive power at 2.4 Hz and has been shown to reduce metabolic power by 16% [12], while a previous study that used an ankle-only exoskeleton with the LN stiffness springs provided 38% of the total positive power at 2.5 Hz and reduced metabolic power by 13% [3]. Some of the differences between the present study and previous studies may stem from differences in hopping biomechanics, as participants in the present study exhibited a 2.5-fold increase in hop height compared with previous studies, which may have altered the MTU contributions during hopping. Additionally, full-leg exoskeleton assistance may influence eccentric muscle contraction and energy storage within series-elastic elements differently to single-joint exoskeletons. Previous investigations have reported that hopping with a passive-elastic ankle-only exoskeleton shifts the average operating length of the plantarflexors to a suboptimal position for producing force economically (onto the ascending arm of the force–length relationship) and increased fascicle excursion and relative shortening velocity during the ground contact phase [4,15]. It is possible that distributing exoskeleton assistance over multiple joints has a different effect on underlying muscle mechanics and allows the muscles to contract at lengths and velocities that are closer to optimal and produce force more economically [27]. Further research is required to explore muscle dynamics with ankle-only and full-leg exoskeletons.

### 4.1. Towards a full-leg running exoskeleton

Similar to hopping, the MTUs surrounding the ankle and knee joints provide the greatest contribution to total average positive power during the stance phase of running [9]. Therefore, a passive-elastic, full-leg exoskeleton with the DG spring stiffness that simultaneously assists multiple joints during the stance phase may also reduce ankle and knee joint positive power while running. However, while the centre-of-mass mechanics in bouncing gaits are similarly described using a spring-mass model, there are differences in joint mechanics between hopping and running that should be considered for future passive-elastic, full-leg running exoskeletons. For example, the passive-elastic, full-leg exoskeleton in the present study used springs that span the entire leg as exoskeleton ‘legs’ and do not allow the knee to flex during the swing phase. Previously, Cherry *et al*. [11] used a friction-lock clutch controlled by participant-specific inputs from thigh velocity and foot switches that enabled a two-piece exoskeleton ‘leg’ to lock during the stance phase and release during the swing phase so that the knee could flex with minimal resistance. Li and Bai [28] developed a novel resolute joint that could produce variable stiffness from pre-tensioning an LN stiffness spring. While not fully passive, future full-leg exoskeletons for level running may benefit from incorporating similar powered elements that allow springs to store and return elastic energy during the stance phase but slacken so that the knee flexes freely during the swing phase and prevent users from working against the spring.

The hip joint also provides a large proportion (32–39%) of the total average positive power during the swing phase when running on level ground at 2.25–3.25 m s^−1^ but does not significantly contribute to positive power during the stance phase [9]. While previous research has shown that passive-elastic assistive devices can reduce the metabolic cost of running by 6.5–8% through hip flexion assistance during the swing phase using tension springs [7,8,29], a passive-elastic, full-leg exoskeleton that acts during the stance phase with compression springs may not significantly contribute to positive hip power. Therefore, minimizing the exoskeleton moment arm about the hip might allow the exoskeleton to assist the ankle and knee during the stance phase without interfering with hip joint mechanics. Additionally, distal mass carried by the legs incurs a metabolic penalty, owing to the increased muscular effort to raise the foot off the ground, move the leg forward during the swing phase and decelerate the leg before the next ground contact [30,31]. The use of lighter-weight materials or redistribution of the exoskeleton mass more proximally would reduce the inertia of the device during the swing phase [11], further reducing the metabolic cost.

Finally, the passive-elastic, full-leg exoskeleton springs are placed laterally to the legs and probably apply a frontal plane moment to the lower-limb joints. In stationary, bilateral hopping, these exoskeleton frontal plane moments are equal and opposite. However, running with one foot on the ground at a time with a passive-elastic, full-leg exoskeleton spring lateral to the leg would apply a frontal plane moment that would need to be counteracted by the user. Thus, users might increase hip abductor moment to help maintain balance, compress the spring and limit frontal plane hip drop during running. A lower profile design that reduces the frontal plane joint moment arms would mitigate these potentially adverse effects.

## 5. Limitations

The exoskeleton used in the present study spans multiple joints and we estimated the exoskeleton’s contribution to joint moment and power using the methods of two previous studies [13,14]. This approach assumed that the influence of the full-leg exoskeleton on each joint was proportional to the external moment arm between the joint centre and exoskeleton line of action and independent of the other joints (e.g. the effect of the exoskeleton at the ankle does not have any additional effects at the knee or hip). In our analyses, we confirmed that summing the exoskeleton contribution to ankle, knee and hip joint power (in all three planes) resulted in a magnitude of power lower than what the springs could provide, indicating that this method did not overestimate the total assistance of the device. However, an alternative approach for determining the exoskeleton’s contribution to joint moment and power might involve conducting inverse dynamics analyses after subtracting the exoskeleton forces from the total vGRF [29] and comparing the differences. Thus, a future study that compares these two methods may be warranted to determine if there is a difference in how exoskeleton assistance is distributed among the joints.

We assumed a rigid connection between the user and the exoskeleton with no soft-tissue displacement. However, it is probable that there was soft-tissue displacement and some energy loss due to the interface between the user and the exoskeleton. Previous studies have identified that user–device interfaces are a significant challenge in developing successful lower-body wearable devices because of the energy lost in soft-tissue displacement [11,32]. Therefore our calculations probably overestimate the exoskeleton contributions to overall joint moments and powers. We tried to mitigate this effect by placing the reflective markers in the same positions on each exoskeleton to minimize the error between exoskeleton trials. Future passive-elastic, full-leg exoskeleton designs may benefit from alternative user interfaces that distribute force over a larger surface area to maximize effective power transfer between the user and the device. Additionally, the mountain bike shoes given to the participants provided an easy method of attaching the exoskeleton legs. However, partially due to the thickness and rigidity of the shoes, we modelled the foot as a single segment and previous studies have shown that this simplification of the foot may overestimate ankle angular displacement (by as much as 10^°^) and ankle power [33]. Participants used the same rigid mountain bike shoes under all conditions, including NH and we assumed the same error in the repeated-measures design to mitigate this potential limitation.

## 6. Conclusions

Hopping using a passive-elastic, full-leg exoskeleton assists both the ankle and knee by reducing the MTU average joint moment and power. Moreover, the magnitude of reductions depends on the spring stiffness profile, where the use of a full-leg exoskeleton with DG spring stiffness provides significantly greater assistance at the ankle than LN or PG spring stiffness, and may explain why previous studies [12] found that the spring stiffness profile within an exoskeleton affects the magnitude of metabolic power reductions. Our data contribute to the increasing number of studies that show that the elastic properties of an exoskeleton spring have a significant effect on hopping mechanics [2,12,18]. Therefore, future studies aiming to develop passive-elastic exoskeletons or investigate the effects of springs in human locomotion should consider the spring stiffness profile.

## Data Availability

Participant average kinematic and kinetic data (N = 14) for each experimental hopping condition and R Markdown scripts to reproduce figures and stats are available to download on the corresponding author's GitHub [34].
